# Late evaluation of upper limb arterial flow in patients after long radial (PiCCO™) catheter placement

**DOI:** 10.1186/s13613-014-0041-9

**Published:** 2015-01-16

**Authors:** Lucas Rovira, Gerardo Aguilar, Alberto Cuñat, Francisco J Belda

**Affiliations:** Anesthesiology and Critical Care Department, Hospital Clínico Universitario, Avd. Blasco Ibañez n°17, Valencia, 46010 Spain; Radiology Department, Hospital Clínico Universitario, Avd. Blasco Ibañez n°17, Valencia, 46010 Spain

**Keywords:** Long radial catheter, PiCCO, Arterial flow, Doppler, ICU, Hemodynamic monitoring

## Abstract

**Background:**

The purpose of the study was to assess blood flow in the upper limb arteries after prolonged catheterization with long radial artery catheters (LRC) which reach the subclavian artery compared to catheterization with standard short radial artery catheters (SRC) and a group of upper limb flow without any catheter placement (NOCATH), with both SRC and NOCATH as control groups.

**Methods:**

Prospective observational study with 20 patients admitted to ICU (40 upper limbs) with LRC and/or SRC inserted >48 h for hemodynamic monitoring. More than 45 days after catheter withdrawal, patients underwent a Doppler ultrasound study of both upper limbs. Arterial flows of arms with LRC (Flow_LRC_) were compared with arterial flows of arms with SRC (Flow_SRC_) and those without any catheter (Flow_NOCATH_).

**Results:**

Flow in the ulnar, brachial, and subclavian arteries did not show any significant difference between the two types of catheters. The only significant difference was in the radial arteries, showing a lower mean flow in the arms with LRC than in the arms with SRC (2.2 vs. 8.5 cc/min; *p* = 0.041). Flow reduction in the radial artery (74%) in the arms with LRC compared to the SRC arms showed a tendency to increase ulnar flow as a compensatory mechanism. None of the patients with LRC included in our study had any ischemic events, in spite of observing complete flow occlusion in three radial arteries (18%) from the Doppler study.

**Conclusions:**

In this sample, the use of PiCCO long radial catheters reaching the subclavian artery did not produce chronic significant changes in brachial or subclavian flows. However, LRC produces a significant reduction in radial flow and a tendency to increase ulnar flow. When comparing these blood flow changes with those produced by SRC use, only the radial flow reduction was significantly lower, whereas the other arterial flow changes did not significantly differ.

## Background

Advanced haemodynamic monitoring is a cornerstone of intensive care [1,2]. Besides the pulmonary artery catheter (PAC), transpulmonary thermodilution (TPTD) is another technology that using a conventional arterial access instead of the pulmonary arterial route avoids several risks and severe complications of the PAC [3] such as arrhythmias and pulmonary arterial embolism. Usually, the femoral arterial access is used for the insertion of the TPTD catheter which, in addition to a conventional lumen providing invasive arterial pressure, includes a thermistor for TPTD. This additional feature slightly increases the diameter compared to a conventional arterial line, being the main reason to prefer femoral arterial access. Despite this, TPTD has also been validated using a special catheter via the radial artery approach [4]. This catheter is 50 cm long in order to reach the subclavian artery and receive a true reflection of central aortic pressure [5]. Due to the increased diameter and length compared to a conventional radial arterial line (4 Fr and 50 cm vs. 2.7 Fr and 8 cm), the radial TPTD arterial line might induce more complications including ischemic events and long-term impairment of blood flow compromising distal perfusion, especially if the collateral circulation is damaged [6].

Most existing studies on arterial patency following these interventions have been conducted to evaluate acute ischemic complications as well as complications of the technique (pseudo-aneurysm, bleeding, and arterial spasms) [7]. However, few studies [8-10] have looked at residual permeability and flow of superior limb arteries after recovery of the patient, and none has been conducted after the use of long radial artery catheters.

We therefore planned a prospective observational study to assess blood flow in the upper limb arteries after prolonged catheterization with long radial artery catheters (LRC group) which reach the subclavian artery. For comparison, we also measured upper limb arterial flow, in one group with prolonged catheterization with standard short radial catheters (SRC group) and in other group without catheterization (NOCATH group). The objective of the study was to know the absolute flow in the arteries of the upper limb 45 days after having used prolonged catheterization with LRC, SRC, or not catheterization and compare them to each other; we also recorded any ischemic event during that time.

## Methods

We designed a prospective study to measure arterial blood flow in the arms of patients after a minimum of 45 days post discharge from the ICU. The study was approved by the ethics committee of the hospital, and eligible patients at discharge from ICU were informed of the study and signed the consent.

The study included patients over 18 years old in whom the long radial PiCCO™ catheter (4 F diameter and 50 cm long) (Pulsion, Munich, Germany), standard short radial catheter (2.7 F and 8 cm long) Arteriofix® (Braun, Germany), or both were used for more than 48 h for the purpose of hemodynamic monitoring.

Catheter insertion was performed under sedation with standard commercial sets following manufacturer indications. Procedures were conducted following the guidelines of the Center for Disease Control (CDC) for the prevention of intravascular catheter-related infections [11]. The arterial catheters were inserted using a modified Seldinger's technique [12,13] by physicians with different degrees of experience. Despite this, no problems were observed during insertion.

In 16 patients, a LRC was primarily inserted for TPTD monitoring. After removing these LRC, in six patients, a SRC was placed in the contralateral arm and in the other ten, no other catheter was placed. Other four different patients were collected in which only SRC were inserted without any other catheter in the contralateral side. This produced an overall of 20 patients in which we measured for the study arterial flows in 16 arms with LRC, 10 arms with SRC, and 14 arms without any catheter. We choose to group all arms by the type of catheter instead of compare flows with their contralateral arm, first, because flow may differ in both arms in healthy people [8] and second, in order to increase the sample size aiming a higher statistical power.

Patients discharged with good outcome from ICU who had signed the consent were contacted more than 45 days later for a Doppler ultrasound study of both upper limbs.

### Doppler study

Doppler ultrasound study was performed to all recruited patients. The study was conducted by an experienced radiologist, skilled in ultrasound, blinded for both location (right or left arm) and type of catheter (long, short, or not). An ultrasound equipment Sonoline Antares™ (Siemens, Erlangen, Germany) was used with 8-MHz linear transducer, Doppler frequency insonation of 4.4 MHz, amplitude between −7 and 2 dB.

All patients underwent blood flow quantification on both upper limbs. The protocol included the analysis of radial, ulnar, brachial, and subclavian arteries. Flow was measured in cc/min. The mean value of measured maximum and minimum flow of each artery was taken as the absolute value for each measurement.

### Data collection and statistics

Demographic data (age, sex, etc.), previous medical of disease (hypertension, diabetes, dyslipidaemia, ischemic heart disease, and others), as well as the reason for ICU admission, type and location of arterial catheter, and duration of catheterization were recorded.

To observe flow, we classified 40 upper limb flows (20 patients) in three groups, limbs in which LRC were inserted (Flow_LRC_), limbs in which short radial catheters (SRC) were inserted (Flow_SRC_), and limbs without any catheter inserted (Flow_NOCATH_).

We additionally recorded any ischemic event during the period of observation and asked for any ischemic symptoms at the time of Doppler study. After at least 45 days following the radial catheters withdrawal, absolute values of arterial flows were measured in both upper limbs.

Flow_LRC_ was compared with Flow_SRC_ or Flow_NOCATH_, and the percentage of change was calculated. This percentage was calculated where Flow_SRC_ or Flow_NOCATH_ was considered to be 100% and compared to Flow_LRC_ applying the formula (Flow_LRC_ × 100 / (Flow_SRC_ or Flow_NOCATH_).

Continuous variables were expressed as median and interquartile range and compared with nonparametric test (*U* Mann–Whitney) as the flows did not follow a normal distribution or *n* < 30.

All *p* values were obtained for two-tailed significance. The statistical test was performed using SPSS 20.0 (IBM, Armonk, New York, USA). Statistical power was calculated *post hoc* by G*Power software (v 3.1.5; Franz Faul, Kiel University, Kiel, Germany).

## Results

We studied a total of 20 patients and 40 upper limbs. Sixteen limbs with LRC (Flow_LRC_), ten limbs with SRC (Flow_SRC_), and fourteen limbs without any catheter (Flow_NOCATH_).

Table 1 shows the demographics of the patients and the main indications for advanced hemodynamic monitoring.

**Table 1:** **Clinical characteristics of the study subjects**

**Clinical characteristics of the study subjects**	
Age (years old)	63 ± 14
Sex	11 men/9 women
BMI	31.23 ± 8.6
HTA	12/20 (60%)
DM type 2	8/20 (40%)
Dislipemia	11/20 (55%)
Heart disease or vasculopathy	10/20 (50%)
Use of antiplatelets or anticoagulants	9/20 (45%)
Days of short radial catheter (Arteriofix®)	5.69 ± 2.41
Days of long radial catheter (PiCCO™)	4.00 ± 4.73
Reason for ICU admission	
Traumatic Brain Injury	6
Cardiovascular surgery	11
Thoracic surgery	2
Sepsis	1

Flow_LRC_ was compared with Flow_SRC_, and both compared with Flow_NOCATH_. Median and interquartile range (IQR) flow in arteries of all these groups are shown in Table 2.

**Table 2:** **Arterial flow; global comparisons**

**Arterial Doppler flows**	**LRC group**	**SRC group**	**NOCATH group**
	**(** ***n*** **= 16)**	**(** ***n*** **= 10)**	**(** ***n*** **= 14)**
**(cc/min)** ^**a**^	**Flow**_**LRC**_	**Flow**_**SRC**_	**Flow**_**NOCATH**_
Ulnar	10.0 (16.0)	6.5 (8)^c^	8.5 (7.5)
	(+53%)^b^	(−23%)	
	(+18%)^c^		
Radial	2.2 (8.7)	8.5 (6.5)	9.2 (15)
	(−74%)^b^; *p* = 0.041	(−8%)^c^	
	(−76%)^c^; *p* = 0.012		
Brachial	43.0 (38.8)	33.5 (20.5)	44.5 (40.9)
	(+28%)^b^	(−25%)^c^	
	(−3%)^c^		
Subclavian	72.0 (65.5)	77.0 (73)	95.5 (64)
	(−6%)^b^	(−19%)^c^	
	(−25%)^c^		

^a^Median (interquartile range); ^b^Compared with Flow_SRC_; ^c^Compared with Flow_NOCATH_. (*p* value <0.05 is significant, and it is shown).

Flow_LRC_ was significantly lower in radial arteries compared with Flow_SRC_ (−74%; *p* = 0.041) and Flow_NOCATH_ (−76%; *p* = 0.012). The rest of upper limb arterial flows did not show significant variations when compared with Flow_SRC_ or Flow_NOCATH_; however, they showed a tendency to increase in the ulnar and brachial arteries and decrease in the subclavian arteries.

When Flow_LRC_ and Flow_SRC_ were compared with Flow_NOCATH_, only the flow variations on the radial arteries were significantly different. Data showed that radial flow was lower in the LRC group (−76%) than in the SRC group (−8%). The rest of the arterial flows did not show significant variations when compared both groups (SRC and LRC) with NOCATH group; at the proximal arteries (brachial and subclavian), the flow reduction occurred in both; at the distal arteries, the ulnar flow only was higher in the LRC group (+18%), being surprisingly lower in the SRC group (−23%).

**Figure 1:**
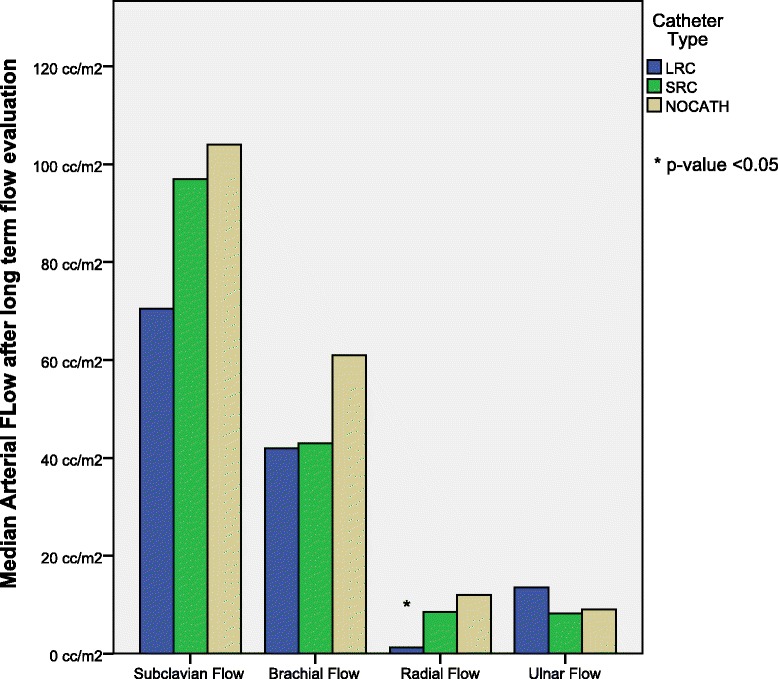
**Median arterial flow after long term evaluation.**

Figure 1 shows a global overview of absolute arterial flow after 45 days, in the arms that have carried LRC, SRC, or NOCATH.

None of the patients with long radial catheters included in our study had an acute ischemic event during their stay in ICU or ischemic symptoms at follow-up evaluation, 45–90 days after catheter withdrawal. However, in the Doppler study, three of these patients (18%) showed complete flow occlusion of the radial artery including one patient who had the longer duration of catheterization (23 consecutive days). The other two patients did not differ from the others in their characteristics. When the number of total occlusions was compared between the LRC group (3/16) with the SRC (0/14), no differences were found (*X*^2^ = 0.088).

## Discussion

This is the first prospective observational study that evaluates upper limb blood flow by Doppler ultrasound, more than 45 days after placement of the long radial PiCCO™ catheter in critically ill patients. When we insert arterial catheters from the PiCCO™ system (for advanced hemodynamic monitoring), the femoral artery is the preferred site, although the radial approach is also validated [4], using arterial catheters which are longer (50 cm) and of higher diameter (4 Fr) than conventional short catheters.

Clinical relevance of using LRC for TPTD is manifest where the use of the radial approach is better than the femoral approach, for example, in patients with coagulation disorders, due to the ease of compression in case of bleeding, in patients with vascular ilio-femoral prosthesis due to the risk of damage, and also in patients where a previous radial cannulation is present, using this previous access. This simplifies the insertion technique avoiding newer punctures and reducing iatrogenia. In our observational study, the main reason to use the radial instead of femoral approach was coagulation disorders and to simplify insertion technique using recent previous radial access (6 of 16 patients with LRC, led a SRC inserted few hours before in the same arm; but for study purpose, due to the limited time that led SRC, only LRC in these arms were considered to be placed).

Endothelial damage is more prevalent after cannulation of the radial artery than the femoral artery [6], which has been attributed to the smaller diameter of the radial artery. This damage in the lumen can cause ischemic injury of the upper limb.

The radial and ulnar arteries provide arterial blood flow to the hand and forearm. The ulnar artery diameter is larger than the radial artery proximally, but distally, at the wrist, this relationship seems to change, and both create a dense anastomotic network of four arches that presents a lot of interindividual anatomical variability [6]. Somehow collateral circulation is normally present. Removal of the radial artery is associated with a significant increase in ulnar artery diameter and blood flow velocity [14]. Harvesting the radial artery for coronary revascularization (a perfect model of radial artery occlusion) appears to be a safe procedure that does not produce upper limb vascular insufficiency of the donor limb because of the increased ulnar flow [15].

In a meta-analysis of short catheters including 4,217 cannulations, ischemic damage of around 20% (6% to 35%) of radial catheter insertions was found [16]. The incidence of temporary radial artery occlusion was 19.7%. Using Doppler ultrasound, authors [17] reported a 24% incidence of complete occlusion 8 days after decannulation; but spontaneous reperfusion occurred progressively over the days, and repermeabilization as late as 75 days after catheter removal may occur [17]. In our study, Doppler flow analysis was conducted more than 45 days after catheter withdrawal allowing spontaneous reperfusion [18]. Therefore, radial artery catheterization is a relatively safe procedure with a permanent ischemic complication rate of only 0.09% as collateral flow usually supplies the radial flow in case of occlusion [16].

In a recent study [3] of long radial artery catheters, insertion of 26 of these catheters produced temporarily pulse loss in only one patient which recovered spontaneously after catheter removal. In our study, no patient in this new series of 16 patients suffered ischemia during their clinical stay, but when analyzing the Doppler arterial flow (>45 days after catheter removal), there were three cases of total occlusion of the radial flow, a percentage (18%) similar to the literature. In all cases, the increased flow in the ulnar artery probably prevented ischemic symptoms.

Globally, we noted that the use of LRC caused a significant reduction in radial flow of 74% (2.2 vs. 8.5 cc/min; *p* = 0.041) when compared with the use of SRC. Ulnar flow tended to increase by around 50%, and despite this, flow was not statistically significant.

More importantly are flow in the brachial and subclavian arteries, because their reduction may induce ischemic damage because no collateral arteries are present. We observed that both flows did not decrease significantly after LRC; while brachial flow showed no reduction, but even increased by 28%. Subclavian flow showed a nonsignificant minimal reduction with LRC when compared with SRC flows.

Subsequently, in order to observe how arterial flow in the upper extremity varies after LRC and SRC use, we compared these flows with the NOCATH group (flow without any catheter placed). We saw that the use of radial arterial catheters caused a chronic reduction in radial flow. This reduction was significantly greater after the use of the long radial artery catheter (−76%; *p* = 0.026) than when using conventional short radial catheters (only −8%; *p* = 0.472). The SRC group did not reduce their radial flow significantly compared with NOCATH group. Additionally, the increase in ulnar flow was greater after LRC (+18%) than SRC. This phenomenon may be related to collaterality effect, in which the greatest reduction in radial flow may induce a greater increase in the ulnar collateral flow [6]. When using LRC, including proximal arteries of the upper limb, the most important fact was that brachial and subclavian flows did not show any statistically significant difference, with a minimal tendency to decrease (−3% and −25%, respectively).

### Limitations

This study was designed as a descriptive pilot study, and the sample size was not predetermined which makes it difficult to obtain statistically relevant conclusions. The *post hoc* analysis of statistical power [19] revealed a power (Error 1-β) of 62% for the *n* used (*n* = 16 vs. *n* = 14), far from the 80% commonly used; at *post hoc* power analysis, we use one-tailed significance test because we only expect radial flow variation in one direction (lower arterial flow with LRC than SRC). *Post hoc* two-tailed significance test was 56% which limits the interpretation of the results.

Despite of the company (Pulsion) recommends that the 50-cm-long radial catheter only be left in place for 3 days, in this observational study, the average length of insertion was 4 ± 4 days due to clinical management requirements; this could increase the cases of total flow occlusion of the radial artery in the LRC group. In the same sense, catheterization was performed by investigators of different experience and skill levels that could produce different degrees of arterial damage at insertion and may influence blood flow in the follow up.

Another limitation lies in the fact that there was no Doppler flow control prior to insertion of any type of catheter, that is to say, paired data before and after cannulation was not available for comparison. In this sense, no conclusive cause-effect relationship can be drawn from our study.

Finally, other studies [8] have shown differences in arterial flows according to the limb-dominance of each person, and we cannot preclude dominance as a confounding factor.

## Conclusions

Use of the long radial PiCCO™ catheter reaching the subclavian artery preserves chronic arterial blood flow to the limb; brachial and subclavian arterial flows were not affected although a significant reduction in radial flow and a tendency to increased ulnar flow as a compensatory mechanism (not statistically significant due to low power analysis) was observed. When comparing these blood flow changes with those produced by short radial catheter use, only the radial flow reduction was significantly lower, whereas ulnar, brachial, and subclavian flow changes did not differ significantly.

Despite finding three cases without radial flow at 45 days, no patient with a long radial catheter showed acute or subacute ischemic events during their stay. Due to the small sample size, this study should be taken as a pilot work for designing more consistent clinical studies with all factors and variables controlled, to establish the absolute safety of using of long radial arterial catheters.

## Abbreviations

PAC: pulmonary artery catheter
TPTD: transpulmonar thermodilution
LRC: long radial catheter
SRC: short radial catheter
NOCATH: without any catheter placed
Flow_LRC_: arterial flows of arms with LRC
Flow_SRC_: arterial flows of arms with SRC
Flow_NOCATH_: arterial flows of arms without any catheter placed
CDC: Center for Disease Control

## References

[1] De Backer D, Fagnoul D, Herpain A (2013). The role of invasive techniques in cardiopulmonary evaluation. Curr Opin Crit Care..

[2] Rajaram SS, Desai NK, Kalra A, Gajera M, Cavanaugh SK, Brampton W (2013). Pulmonary artery catheters for adult patients in intensive care. Cochrane Database Syst Rev..

[3] Belda FJ, Aguilar G, Teboul JL, Pestaña D, Redondo FJ, Malbrain M (2011). Complications related to less-invasive haemodynamic monitoring. Br J Anaesth..

[4] Orme RM, Pigott DW, Mihm FG (2004). Measurement of cardiac output by transpulmonary arterial thermodilution using a long radial artery catheter. A comparison with intermittent pulmonary artery thermodilution. Anaesthesia..

[5] Rulf EN, Mitchell MM, Prakash O, Rijsterborg H, Cruz E, Deryck YL, Rating W, Schepp RM, Siphanto K, Van der Woerd A (1990). Measurement of arterial pressure after cardiopulmonary bypass with long radial artery catheters. J A Cardiothorac Anesth..

[6] Brzezinski M, Luisetti T, London MJ (2009). Radial artery cannulation: a comprehensive review of recent anatomic and physiologic investigations. Anesth Analg..

[7] Jolly SS, Amlani S, Hamon M, Yusuf S, Mehta SR (2009). Radial versus femoral access for coronary angiography or intervention and the impact on major bleeding and ischemic events: a systematic review and meta-analysis of randomized trials. Am Heart J..

[8] Davis FM, Stewart JM (1980). Radial artery cannulation. A prospective study in patients undergoing cardiothoracic surgery. Br J Anaesth.

[9] Bedford RF (1978). Long-term radial artery cannulation: effects on subsequent vessel function. Crit Care Med..

[10] Eker HE, Tuzuner A, Yilmaz AA, Alanoglu Z, Ates Y (2009). The impact of two arterial catheters, different in diameter and length, on postcannulation radial artery diameter, blood flow, and occlusion in atherosclerotic patients. J Anesth.

[11] Boyce JM, Pittet D, Healthcare Infection Control Practices Advisory Committee, HICPAC/SHEA/APIC/IDSA Hand Hygiene Task Force (2002). Guideline for hand hygiene in health-care settings: recommendations of the Healthcare Infection Control Practices Advisory Committee and HICPAC/SHEA/APIC/IDSA Hand Hygiene Task Force. MMWR Morb Mortal Wkly.

[12] Seldinger S (1953). Catheter replacement of the needle in percutaneous arteriography; a new technique. Acta Radiol..

[13] Mangar D, Thrush DN, Connell GR, Downs JB (1993). Direct or modified Seldinger guide wire-directed technique for arterial catheter insertion. Anesth Analg..

[14] Brodman RF, Hirsh LE, Frame R (2002). Effect of radial artery harvest on collateral forearm blood flow and digital perfusion. J Thorac Cardiovasc Surg..

[15] Schena S, Crabtree TD, Baker KA, Guthrie TJ, Curci J, Damiano RJ, Barner HB (2011). Absence of deterioration of vascular function of the donor limb at late follow-up after radial artery harvesting. J Thorac Cardiovasc Surg..

[16] Scheer B, Perel A, Pfeiffer UJ (2002). Clinical review: complications and risk factors of peripheral arterial catheters used for haemodynamic monitoring in anaesthesia and intensive care medicine. Crit Care..

[17] Bedford RF, Wollman H (1973). Complications of percutaneous radial-artery cannulation: an objective prospective study in man. Anesthesiology..

[18] Doscher W, Viswanathan B, Stein T, Margolis IB (1983). Hemodynamic assessment of the circulation in 200 normal hands. Ann Surg..

[19] Faul F, Erdfelder E, Lang A, Buchner A (2007). G*Power 3: a flexible statistical power analysis program for the social, behavioral, and biomedical sciences. Behav Res Methods..

